# Energy absorption ability of buckyball C_720_ at low impact speed: a numerical study based on molecular dynamics

**DOI:** 10.1186/1556-276X-8-54

**Published:** 2013-01-29

**Authors:** Jun Xu, Yibing Li, Yong Xiang, Xi Chen

**Affiliations:** 1 Columbia Nanomechanics Research Center, Department of Earth and Environmental Engineering, Columbia University, New York, NY 10027, USA; 2 State Key Laboratory of Automotive Safety and Energy, Department of Automotive Engineering, Tsinghua University, Beijing, 100084, People’s Republic of China; 3 State Key Lab of Electronic Thin Films and Integrated Devices, School of Energy Science and Engineering, University of Electronic Science and Technology of China, Chengdu, Sichuan 611731, People’s Republic of China; 4 Department of Civil and Environmental Engineering, Hanyang University, Seoul, 133-791, South Korea; 5 International Center for Applied Mechanics, SV Lab, Xi’an Jiaotong University, Xi’an, 710049, People’s Republic of China

**Keywords:** Impact, Energy absorption, Buckyball, Buckling

## Abstract

The dynamic impact response of giant buckyball C_720_ is investigated by using molecular dynamics simulations. The non-recoverable deformation of C_720_ makes it an ideal candidate for high-performance energy absorption. Firstly, mechanical behaviors under dynamic impact and low-speed crushing are simulated and modeled, which clarifies the buckling-related energy absorption mechanism. One-dimensional C_720_ arrays (both vertical and horizontal alignments) are studied at various impact speeds, which show that the energy absorption ability is dominated by the impact energy per buckyball and less sensitive to the number and arrangement direction of buckyballs. Three-dimensional stacking of buckyballs in simple cubic, body-centered cubic, hexagonal, and face-centered cubic forms are investigated. Stacking form with higher occupation density yields higher energy absorption. The present study may shed lights on employing C_720_ assembly as an advanced energy absorption system against low-speed impacts.

## Background

Absorption of external impact energy has long been a research topic with the pressing need from civil [1,2] to military needs [3,4]. In particular, effective absorption of mechanical energy at low-impact speed, i.e., below 100 m/s is of great interest [5,6]. As one of the major branches of fullerene family, the carbon nanotube (CNT) has demonstrated an outstanding mechanical energy dissipation ability through water-filled CNT [7], CNT forest and bundle [7], CNT/epoxy nanocomposites [8], CNT immersed in nonaqueous liquid [9], intercalating vertical alignment with aligned existing layered compounds [10], and sponge-like material containing self-assembled interconnected CNT skeletons [11], among others. The advantage lies within the CNTs’ intriguing mechanical properties, i.e., ultra-strong (Young’s modulus of 0.9 to 5.5 TPa [12-14] and tensile strength of 60 GPa [12]) and ultra-light, as well as the tube structure which buckles upon external loadings [15]. Both theoretical modeling [16-18] and experiments [19-21] have proposed that the energy dissipation density of CNTs could be on the order of 200 J/cm^3^, about 1-2 order of magnitudes over traditional engineering material [1].

Naturally, another branch of fullerene family with a spherical shape, i.e., the buckyball, also possesses excellent mechanical properties similar to CNTs. Man et al. [22] examined a C_60_ in collision with a graphite surface and found that the C_60_ would first deform into a disk-like structure and then recover to its original shape. It is also known that C_60_ has a decent damping ability by transferring impact energy to internal energy [23,24]. This large deformation ability under compressive strain of C_60_ was also verified by Kaur et al. [25]. For higher impact energy, Zhang [26] employed C_60_/C_320_ to collide with mono/double layer graphene, and the penetration of graphene and the dissociation of buckyball were observed. Furthermore, Wang and Lee [27] observed a novel phenomenon of heat wave propagation driven by impact loading between C_60_ and graphene which was responsible for the mechanical deformation of the buckyball. Meanwhile, giant buckyballs, such as C_720_, have smaller system rigidity as well as non-recoverable morphology upon impact, and thus they are expected to have higher capabilities for energy dissipation [28]. However, to the best knowledge of the authors, currently, only few studies about the mechanical behavior of giant buckyball are available [29-31].

To understand the mechanical behavior of C_720_ and investigate its energy absorption potential in this paper, the dynamic response of C_720_ is studied at various impact speeds below 100 m/s by employing molecular dynamics (MD) simulations. Firstly, the buckling behaviors under both low-speed crushing and impact are discussed and described using classical thin shell models. Next, 1-D alignment of C_720_ system is investigated to identify the influence of packing of the buckyball on unit energy absorption. Finally, 3-D stacking of C_720_ system is considered, where four types of packing forms are introduced and the relationship between unit energy absorption and stacking density are elucidated by an empirical model.

## Methods

### Computational model and method

**Figure 1:**
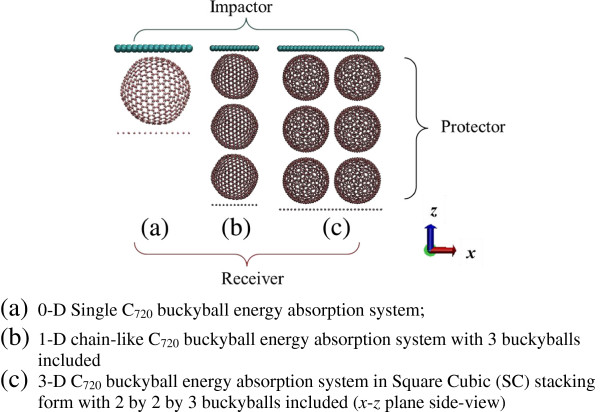
Various alignments of buckyball system as a protector.

The C_720_ is a spherical buckyball with diameter of 2.708 nm (where the van der Waals equilibrium distance is considered), volume of 7.35 nm^3^, and mass of 1.45 × 10^−20^ g. C_720_ with varying numbers and packing directions (both vertical and horizontal) are selected in this study. Computational cells from single buckyball to 3-D buckyball stacking system are illustrated in selected examples in Figure 1. In the scenario of the impact, the buckyball system subjects to the impact of a top rigid plate with incident energy *E*_impactor_, and the initial impact speed is below 100 m/s; in the scenario of crushing, the top rigid plate compresses the buckyball system at a constant speed below 100 m/s. The bottom plate, which is rigid and fixed, serves as a receiver, and the force history it experiences could indicate the energy mitigation capability of the protective buckyball system. The buckyball is not allowed to slip with respect to the impactor and receiver plates. Both the impactor and receiver plates are composed of carbon atoms. The masses of the atoms are varied in the following simulation to set various loading conditions (varying impactor mass), while the interactions between the plates and buckyballs remain as carbon-carbon interaction.

MD simulation is performed based on large-scale atomic/molecular massively parallel simulator platform with the micro-canonical ensembles (NVE) [32] after equilibration. A pairwise Lennard-Jones (L-J) potential term is added to the buckyball potential to account for the steric and van der Waals carbon-carbon interaction

(1) $$U\left(r_{\text{ij}}\right)=4\varepsilon_{\text{CC}}\left[{\left(\frac{\sigma_{\text{CC}}}{r_{\text{ij}}}\right)}^{12}-{\left(\frac{\sigma_{\text{CC}}}{r_{\text{ij}}}\right)}^{6}\right]$$

where *ɛ*_CC_ is the depth of the potential well between carbon-carbon atoms, *σ*_CC_ is the finite distance where the carbon-carbon potential is zero, *r*_*ij*_ is the distance between the two carbon atoms. Here, L-J parameters for the carbon atoms of the buckyball $\sigma_{\text{CC}}=3.47\dot{A}$ and *ε*_CC_ = 0.27647 kJ/mol as used in the original parametrization of Girifalco [33] and van der Waals interaction govern in the plate-buckyball interaction. A time integration step of 1 fs is used, and periodical boundary conditions are applied in the *xy* plane to eliminated the boundary effect.

### Single buckyball mechanical behavior

#### Atomistic simulation result

**Figure 2:**
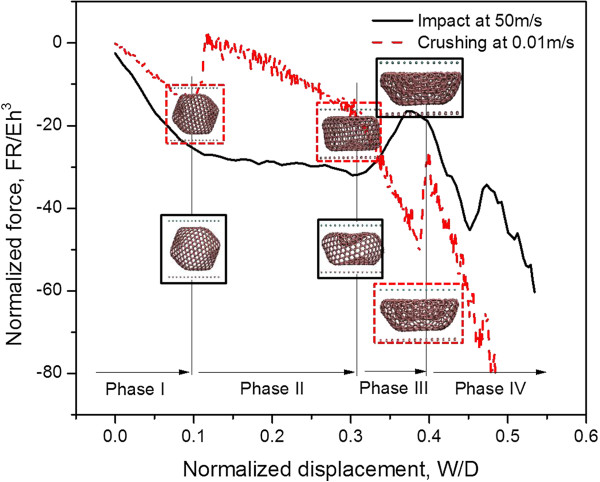
**Normalized force displacement curves at both low-speed crushing and impact loading.** The entire process from the beginning of loading to the bowl-forming morphology can be divided into four phases. Morphologies of C_720_ are shown at the corresponding normalized displacements.

The distinctive mechanical behavior of a single buckyball should underpin the overall energy absorption ability of a buckyball assembly. The force *F* and displacement *W* are normalized as *FR/Eh*^3^ and *W*/*D*, respectively, where *R*, *h*, *D*, and *E* are the radius, effective thickness, diameter, and effective Young’s modulus of the buckyball, respectively. Considering that bending is involved during the buckyball compression, *h* = 0.66 nm and *E* = 5 TPa [34,35]. Here a crushing speed at 0.01 m/s is employed to mimic quasi-static loading, because the normalized force-displacement curves are verified to be the same at various loading rates from 0.1 to 0.001 m/s in trial simulations. The force-displacement response under both quasi-static and a representative dynamic impact loading (with impact speed of 50 m/s and energy of 1.83 eV) are studied, as shown in Figure 2. Two obvious force-drops could be observed in low-speed crushing, while only one prominent force-drop exists in dynamic loading which is related to the less-evident snap-through deformation shape.

The entire compression process could be divided into four phases according to the *FR*/*Eh*^3^ ~ *W*/*D* curve, i.e., buckling (*W*/*D* < 10%), post-buckling (10% ≤ *W*/*D* < 30%), densification (30% ≤ *W*/*D* < 40%), and inverted-cap-forming phase (*W*/*D* > 40%). Upon the ricochet of the plate, the deformation remains as a bowl shape with great volume shrinkage. The stabilization of such a buckled morphology is owing to a lower system potential energy in the buckled configuration due to van der Waals interaction; similar energy dissipation mechanism in CNT network is also revealed by [36].

The derivative of curve undergoes a sudden change at the same *W*/*D* value but in two completely different loading rates, suggesting that the sudden force-drop points are highly dependent on the buckyball deformation rather than the loading rate. And theoretical insights may be obtained from the four-phase deformation.

#### Phenomenological mechanical models

Note that due to the property of *FR*/*Eh*^3^ ~ *W*/*D* curve, among the phases of compression process, those with significant reduction of force (Figure 2) are relatively unimportant for energy absorption and not included in the modeling effort. A three-phase model for low-speed crushing and a two-phase model for impact loading are proposed separately in the following sections.

#### Three-phase model for low-speed crushing (quasi-static loading)

(1) Phase I. Buckling phase

**Figure 3:**
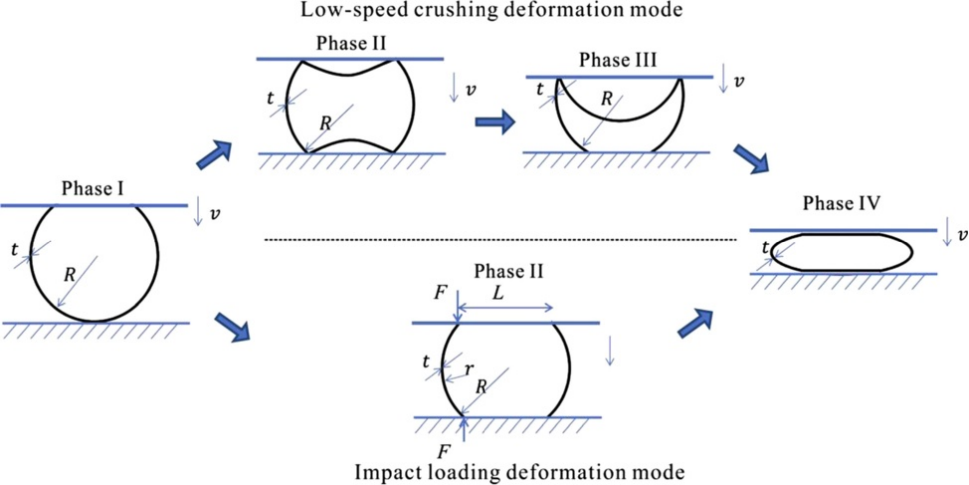
**Illustration of deformation phases during compression for C**_**720**_**.** Dynamic loading and low-speed crushing share the same morphologies in phase I while they are different in phase II. Analytical models are based on the phases indicated above and below the dash line for low-speed crushing and impact loading, respectively.

In the range of small deformation in the beginning of compression, the model describing thin-shell deformation under a point force is applicable [37,38]. Considering a buckyball with wall thickness *h* = 0.066 nm compressed by *F* with deformation of *W* (with the subscript number denoting the phase number sketched in Figure 3), the force-deflection relation should be expressed as [39]

(2) $$F_{1}=\frac{8G}{\text{Rc}}W_{1}\;\left(0<W_{1}\leq W_{b1}\right)$$

where the bending stiffness *G* = *Ehc*^2^; the reduced wall thickness $c=h/\sqrt{12\left(1-\nu^{2}\right)}$ and *ν* is the Poisson’s ratio. The linear deformation behavior continues until it reaches the critical normalized strain *W*_b1_. Experimental results for bulk thin spherical shell show that the transition from the flattened to the buckled configuration occurs at a deformation close to twice the thickness of the shell [40]; while *W*_b1_ here is about 4 *h*, indicating a larger buckling strain in nanoscale structure.

The nanostructure has higher resistance to buckle than its continuum counterpart and based on the fitting of MD simulation, a coefficient f^*^ ≈ 2.95 should be expanded to Equation 2 as

(3) $$F_{1}=\frac{8G}{\text{Rc}}W_{1}\cdot \left(f^{*}\right)\left(0<W_{1}\leq W_{b1}\right)$$

It is reminded that this equation is only valid for C_720_ under low-speed (or quasi-static) crushing.

(2) Phase II. Post-buckling phase

As the compression continues, buckyball continues to deform. Once the compressive strain reaches *W*_b1_, the flattened area becomes unstable and within a small region, the buckyball snaps through to a new configuration in order to minimize the strain energy of the deformation, shown in Figure 3. The ratio between the diameter and thickness of buckyball is quite large, i.e., *D*/*h* ≈ 36.5, and only a small portion of volume is involved thus the stretching energy is of secondary order contribution to the total strain energy. Hubbard and Stronge [41] developed a model to describe the post-buckling behavior of a thin spherical shell under compression based on Steele’s [42] model

(4) $$F_{2}=\sqrt{\frac{2W_{2}h}{K}}\frac{16G}{\text{Rc}}\left(W_{b1}<W_{2}\leq W_{b2}\right)$$

where $K={\left(\frac{5}{4}\right)}^{2}{\left(\frac{8}{3\pi }\right)}^{2}\sqrt{3\left(1-\nu^{2}\right)}$. This nonlinear deformation behavior extends until it reaches the densification critical normalized strain *W*_b2_. The value of *W*_b2_ could be fitted from the simulation data for C_720_ where *W*_b2_ ≈ 11*h*.

The first force-drop phenomenon is obvious once the buckling occurs where the loading drops to nearly zero. Therefore, by applying the boundary condition of *F*_2_(*W*_2_) ≈ 0, Equation 4 maybe further modified as

(5) $$F_{2}=\sqrt{\frac{2h}{K}}\frac{16G}{\text{Rc}}\;\left(\sqrt{W_{2}}-\sqrt{W_{b2}}\right)\cdot \left(f^{*}\right)\;\left(W_{b1}<W_{2}\leq W_{b2}\right)$$

(3) Phase III. Densification phase

When the compression goes further, the crushing displacement eventually becomes much larger than the thickness and thus the force-displacement relation becomes nonlinear [42]. The buckled buckyball is densified during this process. A phenomenological nonlinear spring-like behavior could be fitted as

(6) $$F_{3}=\gamma W_{3}^{n}\text{,}$$

where *γ* is a coefficient and *n* is fitted as *n* ≈ 1.16. Considering the relationship [41,42]

(7) $$\frac{2W_{3}}{h}=K{\left(\frac{F_{b}}{2}\right)}^{n}$$

and

(8) $$F_{b}=\frac{F_{3}R}{8G}\frac{c}{h}\text{,}$$

we may come to the equation

(9) $$F_{3}=\frac{16\text{Gh}}{\text{Rc}}{\left(\frac{2}{\text{Kh}}\right)}^{n}W_{3}^{n}\;\left(W_{b2}<W_{3}\right)$$

Thus, by considering the continuity of two curves in adjacent phases, we may rewrite Equation 9 as

(10) $$F_{3}=\left(\frac{16\text{Gh}}{\text{Rc}}{\left(\frac{2}{\text{Kh}}\right)}^{n}\left(W_{3}^{n}-W_{b2}^{n}\right)+F_{2}\left(W_{b2}\right)\right)\cdot \left(f^{*}\right)\left(W_{b2}<W_{3}\right)$$

Therefore, Equations 3, 5, and 10 together serve as the normalized force-displacement model which may be used to describe the mechanical behavior of the buckyball under quasi-static loading condition from small to large deformation.

**Figure 4:**
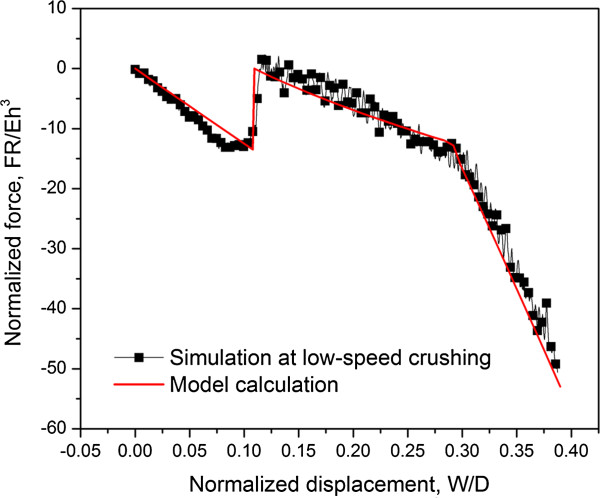
Comparison between computational results and analytical model at low-speed crushing of 0.01 m/s.

Figure 4 shows the simulation data at low-speed crushing compared with the model calculation. A good agreement between two results is observed which validates the effectiveness of the model.

#### Two-phase model for impact

The mechanical behaviors of buckyball during the first phase at both low-speed crushing and impact loadings are similar. Thus, Equation 2 is still valid in phase I with a different f^*^ ≈ 4.30. The characteristic buckling time, the time it takes from contact to buckle, is on the order of *τ* ≈ 10^− 1^ ~ 10^0^ ns ~ *T* ≈ 2.5*R*/*c*_1_ ≈ 5.71 × 10^− 5^ns, where *ρ* is the density of C_720_ and $c_{1}=\sqrt{E/\rho }$. It is much longer than the wave traveling time; thus, the enhancement of f^*^ should be caused by the inertia effect [43].

As indicated before, the buckyball behaves differently during the post-buckling phase if it is loaded dynamically, i.e., no obvious snap through would be observed at the buckling point such that the thin spherical structure is able to sustain load by bending its wall. Therefore, a simple shell bending model is employed here to describe its behavior as shown in Figure 3; the top and bottom flattened wall with length of *L* experiences little stretching strain, whereas the side wall bends with finite deformation, governing the total system strain energy

(11) $$E_{\text{system}}=\frac{1}{2}\int_{A}\frac{M^{2}}{\text{EI}}dA$$

where the bending rigidity $\text{EI}=\frac{E{h^{\prime }}^{3}}{12\left(1-\nu^{2}\right)}$ and *M* is the bending moment. *A* denotes the integration area. The *h*^’^ is the ‘enlarged’ thickness, the result of smaller snap-through phenomenon. Here, *h*^’^ ≈ 1.40*h* via data fitting. Substituting geometrical constraints and taking the derivative, the force-displacement relation becomes (for C_720_ under 100 m/s impact)

(12) $$F_{2}=\frac{1}{4}\text{EI}\frac{1}{{\left(R-W_{2}/2\right)}^{2}}\pi^{2}\cdot \mathrm{\pi R}\;\left(W_{b1}<W_{2}\leq W_{b2}\right)$$

Therefore, Equations 3 and 12 together provide a model to describe the mechanical behavior of the buckyball under dynamic loadings.

**Figure 5:**
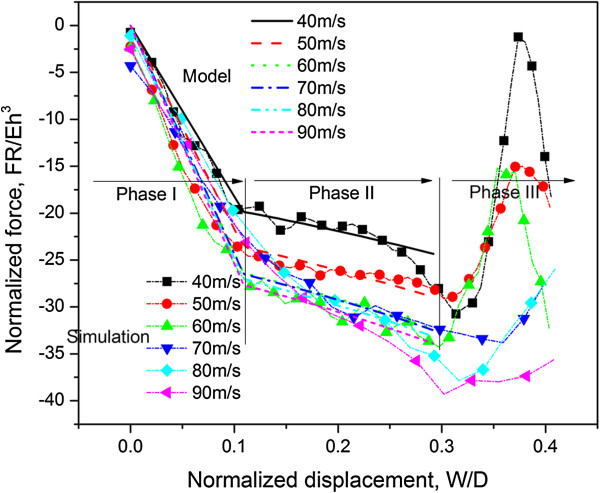
**Comparison between computational results and analytical model.** Comparison between computational results and analytical model at various impact speeds from 40 to 90 m/s.

When the impact speed is varied, the corresponding force is modified by a factor *α* owing to strain rate effect [44-46]. With the subscript representing the impact speed (in units of m/s), the correction factor **c** = *α*_40_, *α*_50_, *α*_60_, *α*_70_, *α*_80_, *α*_90_ = [0.83, 1.00, 1.12, 1.14, 1.17]. Figure 5 illustrates the comparison between atomistic simulation and model (for impact speeds of 40 to 90 m/s), with good agreements.

## Results and discussion

### Buckyball assembly

In practice, buckyballs need to be assembled (shown in Figure 1) so as to protect materials/devices. Various stacking arrays are investigated as follows.

#### 1-D alignment buckyball system

**Figure 6:**
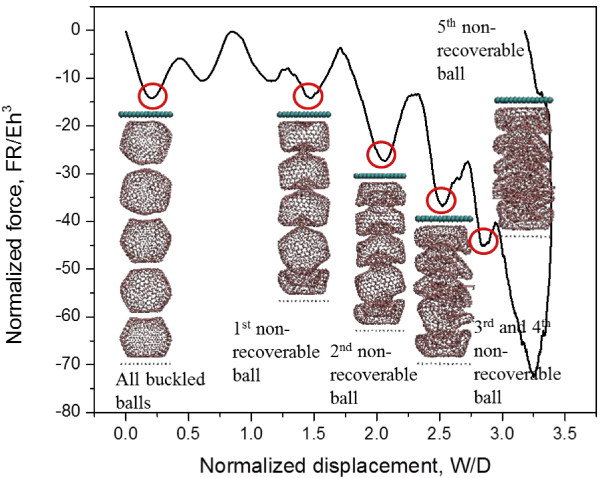
**Characteristic normalized force-displacement curve of 1-D system with vertically lined C**_**720**_**buckyballs.** The characteristic normalized force-displacement curve of 1-D system with five vertically lined C_720_s at impact speed of 50 m/s.

The C_720_ can be arranged both vertically and horizontally in a 1-D chain-like alignment. Figure 6 shows the mechanical behavior of a five-buckyball array subjecting to a rigid plate impact with impact energy and speed of 9.16 eV and 50 m/s respectively. Progressive buckling and bowl-shape forming behavior takes the full advantage of single buckyball energy absorption ability one by one and controls the force on the receiver within a relatively low value during first section of deformation (within *W*/*D* < 1.5) which provides cushion protections.

**Figure 7:**
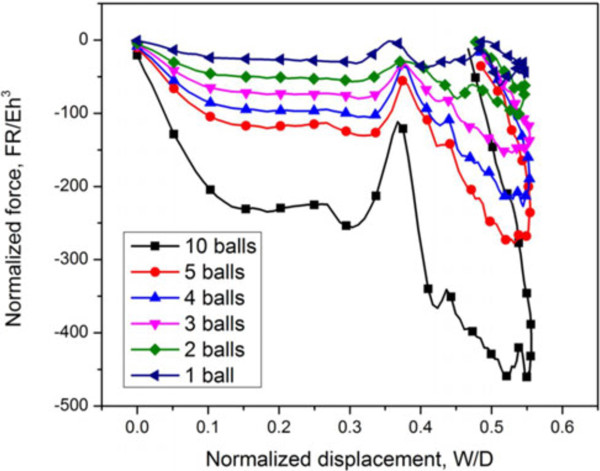
**Characteristic normalized force-displacement curve of 1-D buckyball system with various numbers of horizontally lined C**_**720**_**buckyballs.** The characteristic normalized force-displacement curve of 1-D buckyball system with various numbers of horizontally lined C_720_s at impact speed of 50 m/s.

Another 1-D arrangement direction is normal to a plate impact. Unlike the progressive buckling behavior in the vertical system, all buckyballs buckle simultaneously in the horizontal array. Figure 7 shows the scenario with impact energy of 1.83 eV per buckyball and impact speed of 50 m/s, where the total reaction force scales with the number of buckyballs. Systems with different buckyball numbers show almost uniform deformation characteristics of individual buckyballs.

**Figure 8:**
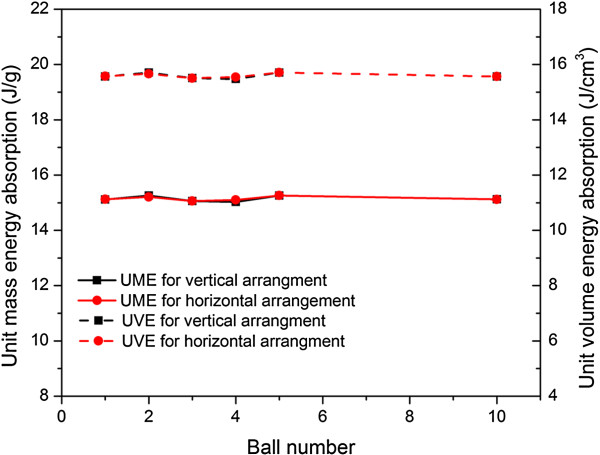
**UME and UVE values of both vertical and horizontal buckyball systems with various buckyball numbers.** UME and UVE values of both vertical and horizontal buckyball systems with various buckyball numbers at impact speed of 50 m/s.

The energy absorption per unit mass (UME, J/g) and unit volume (UVE, J/cm^3^) are given in Figure 8, which shows that the UME and UVE are almost invariant regardless of buckyball number or arrangement. In Figure 8 the impact energy per buckyball is fixed as 1.83 eV; if the impact energy or speed changes, the value of UME or UVE alters; however, the result is still insensitive to buckyball number or arrangement. The major responsible reason is that the energy absorption ability of the system stems from the non-recoverable deformation of individual buckyball which is almost uniform.

**Figure 9:**
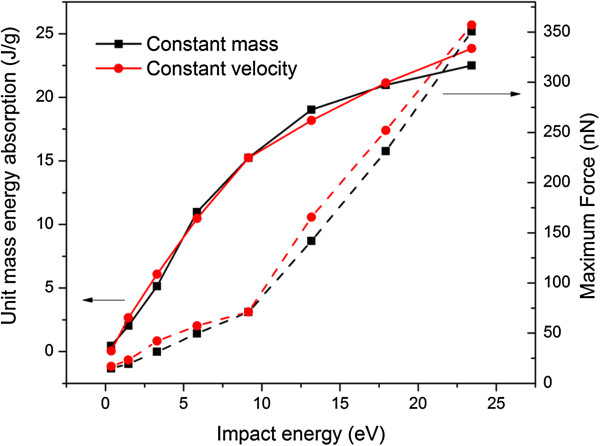
**UME and maximum contact force at constant impact speed (50 m/s) with various impact masses.** UME and maximum contact force at constant impact speed (50 m/s) with various impact mass (from 8.7 × 10^−19^ to 7.1 × 10^−17^ g), and constant impact mass (2.8 × 10^−18^ g) with various impact speeds (from 10 to 90 m/s), for five-buckyball systems.

By fixing either the impact speed or mass and varying the other parameter, the impact energy per buckyball can be varied. It imposes a nonlinear influence on the UME and the maximum force on the receiver, as shown in Figure 9 for the vertical alignment of five-buckyball system. No matter how the impact speed or mass varies, it is the impact energy per buckyball that dominates the values of UME and maximum transmitted force.

#### 3-D stacking buckyball system

The packing density of a 3-D stacking system can be different than that of the 1-D system, and thus the performance is expected to vary. Four types of 3-D stacking forms are investigated, i.e., simple cubic (SC), body-centered cubic (BCC), face-centered cubic (FCC) (a basic crystal structure of buckyball [47]), and hexagonal-closed packing (HCP). The occupation density *η* _*SC*_ = *π*/6 ≈ 0.52, $\eta_{\text{BCC}}=\pi \sqrt{3}/8\approx 0.68$, $\eta_{\;\text{FCC}}=\eta_{\;\text{HCP}}=\pi /3\sqrt{2}\approx 0.74$[48] for SC, BCC, FCC, and HCP, respectively. Convergence study indicates that the profiles of force-displacement curves as well as the energy absorption rate at increasing buckyball numbers at one computational cell keep the same. In this case, a fundamental unit, such as containing 2 × 2 × 3 buckyballs for SC arrangement is shown in Figure 1c.

**Figure 10:**
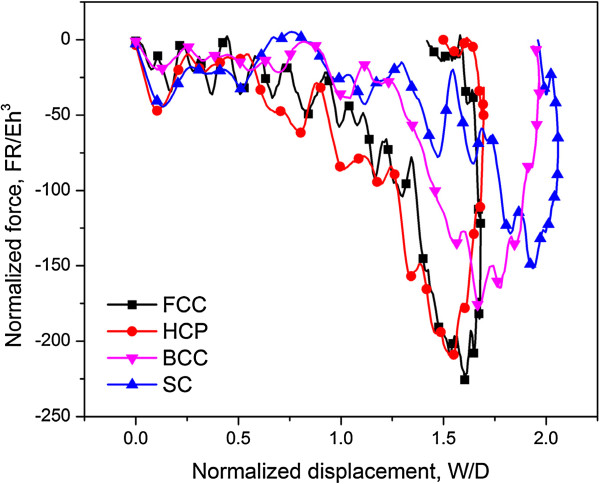
**Normalized force-displacement curves for SC, BCC, FCC and HCP packing of C**_**720**_**.** Typical normalized force-displacement curves for SC, BCC, FCC and HCP packing of C_720_ at impact speed of 50 m/s, and the impact energy per buckyball is 1.83 eV.

Figure 10 illustrates the normalized force-displacement curves for SC, BCC, FCC, and HCP units under the same impact energy per buckyball (1.83 eV). As expected, the mechanical behaviors of FCC and HCP are similar, while the BCC and SC units (with lower *η*) have more space for system to comply and hence the impact force is smaller yet the displacement is larger. Consequently, FCC and HCP have the same energy absorption ability and that of BCC and SC are inferior.

**Figure 11:**
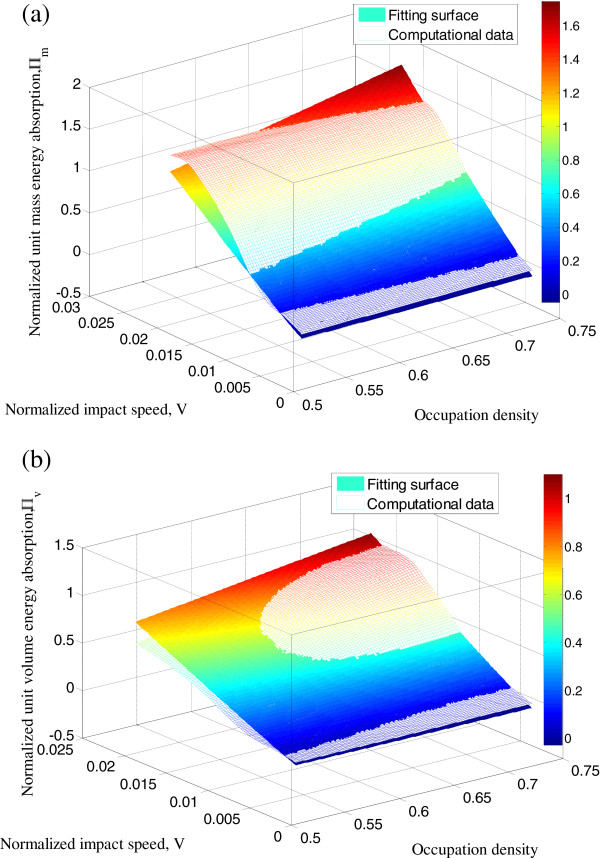
**UME and UVE values of SC, BCC, FCC, and HCP packing of C**_**720 **_**at impact speeds.** UME and UVE values of SC, BCC, FCC, and HCP packing of C_720_ at impact speeds from 10 m/s to 90 m/s. Fitting surfaces based on the empirical equations are also compared with the simulation. (**a**) UME values of various packing forms of C_720_ at impact various impact speeds. (**b**) UVE values of various packing forms of C_720_ at impact various impact speeds.

Energy absorption performances of the three basic units are studied at various impact speeds, i.e., from 10 to 90 m/s while the impact mass is kept a constant, as shown in Figure 11. With the impact speed increases, more mechanical energy is absorbed; but the increasing trend becomes slighter at higher impact speed when the buckyball system reaches its mitigation limit. The improvement is greater in terms of UVE than UME with higher *η*.

By normalizing the UME and UVE as *Π*_m_ = UME/(*E*_impactor_/*m*) and *Π*_*v*_ = UVE/(*E*_impactor_/*V*_volume_) where *V*_volume_ is the volume of the buckyball and impact speed as $V=v/\sqrt{B/\rho }$ where *B* = 34 GPa [49] is the bulk modulus of graphite. An empirical equation could be fitted as

(13) $$\Pi_{m}=\mathrm{A\eta}\left(BV^{C}+\text{DV}\right)$$

where *A* = 5.50, *B* = −0.25, *C* = 0.21, and *D* = 25.0 with fitting correlation coefficient of 0.96 and

(14) $$\Pi_{v}=\mathrm{A\eta}\left(BV^{C}+\text{DV}\right)\text{,}$$

where *A* = 0.46, *B* = −1.94, *C* = 0.21, and *D* = 187.9 with fitting correlation coefficient of 0.96. These equations are valid for low-speed impact speed (below 100 m/s) on stacked C_720_ buckyballs. When the impact speed is fixed, the unit energy absorption linearly increases with the occupation density; under a particular spatial arrangement, the energy absorption ability increases nonlinearly with the impact speed.

## Conclusions

C_720_ as a representative giant buckyball has the distinctive property of non-recovery deformation after crushing or impact, which makes it capable of absorbing a large amount of energy. The mechanical behaviors of a single C_720_ under quasi-static (low-speed crushing) and dynamic impact are investigated via MD simulation and analytical modeling. By understanding the mechanism of mechanical behavior of individual C_720_, the energy absorption ability of a 1-D array of buckyball system is studied. It is found that regardless of the direction of alignment and number of buckyballs, the unit energy absorption density is almost the same for low-speed impact. In addition, different 3-D stacking at various impact speeds and stacking forms are investigated. Explicit empirical models are suggested where packing density and impact speed may pose a positive effect on the unit energy absorption. This study may shed lights on the buckyball dynamic mechanical behavior and its application in energy absorption devices and inspire the related experimental work.

## Competing interests

The authors declare that they have no competing interests.

## Authors’ contributions

JX carried out the molecular dynamic simulation and drafted the manuscript. YL participated in the design of the study and performed the mechanical analysis. XC and YX conceived of the study and participated in its design and coordination and helped draft the manuscript. All authors read and approved the final manuscript.

## Authors’ information

JX is a Ph.D. candidate in Department of Earth and Environmental Engineering at Columbia University, supported by the Presidential Distinguished Fellowship. His research interests are nanomechanics and energy-related materials. YL is a Professor in Department of Automotive Engineering at Tsinghua University. He has been awarded by the National Science and Technology Advancement Award (second prize) for twice. His major research interests are advanced energy absorption material. YX is a Professor in School of Energy Science and Engineering at University of Electronic Science and Technology of China. His research is focused on combinatorial materials research with emphasis on energy applications, particularly on thin film materials and devices, printed electronics, and power electronics. He has authored and co-authored more than 40 articles, with an *h*-index of 12. XC is an Associate Professor in Department of Earth and Environmental Engineering at Columbia University. He uses multiscale theoretical, experimental, and numerical approaches to investigate various research frontiers in materials addressing challenges in energy and environment, nanomechanics, and mechanobiology. He has published over 200 journal papers with an *h*-index over 30.
